# Combined stomach content and stable isotope analyses revealed variations in trophic ecology of pacific cod in the east sea

**DOI:** 10.1038/s41598-025-20151-1

**Published:** 2025-10-17

**Authors:** Min Gu Kang, Samroz Majeed, Hae Kun Jung, Chung Il Lee, Hyun Je Park, Jong Won Park, Hyun Soo Rho, Won Gi Min, Joo Myun Park

**Affiliations:** 1 East Sea Environment Research Center, Korea Institute of Ocean Science & Technology, Uljin, 36315 Republic of Korea; 2 Department of Ocean Science, University of Science & Technology, Daejeon, 34133 Republic of Korea; 3 Ocean Climate and Ecology Research Division, National Institute of Fisheries Science, Busan, 46083 Republic of Korea; 4 Department of Marine Bioscience, Gangneung-Wonju National University, Gangneung, 25457 Republic of Korea; 5 Department of Marine Biology, Kunsan National University, Gunsan, 54150 Republic of Korea

**Keywords:** Pacific cod, Stomach content, Stable isotope, Trophic level, East sea, Ecology, Zoology, Ecology, Ocean sciences

## Abstract

This study investigated changes in the dietary composition and trophic levels of Pacific cod (*Gadus macrocephalus*) using stomach content and stable isotope analyses. Pacific cod samples were collected seasonally from May 2018 to February 2022 using bottom gill nets at the northern (Ayajin) and southern (Hupo) sites of the East Sea along the Korean coast. The primary dietary components included teleosts, amphipods, carid shrimps, and cephalopods. Gravimetric dietary analysis revealed distinct latitudinal, seasonal, and size-related variations, with teleosts, cephalopods, carid shrimps, and euphausiids serving as the key prey items. During the cold season, teleosts constituted the major dietary components of the large-sized Pacific cod, whereas smaller individuals consumed more carid shrimps, amphipods, and euphausiids. In the warm season, large-sized individuals predominantly consumed cephalopods irrespective of location, whereas smaller individuals favored amphipods and euphausiids at Ayajin and carid shrimps at Hupo. Differences in stable isotope values (δ^13^C and δ^15^N) between Ayajin and Hupo, between size classes, indicated higher values in the Hupo and in large-sized individuals. Increased consumption of teleosts and cephalopods by larger Pacific cod led to enriched isotope values, whereas the contribution of carid shrimps elevated the trophic level at Hupo. This study provides comprehensive insights into the dietary variations and trophic levels of Pacific cod in response to changes in prey availability.

**Supplementary Information:**

The online version contains supplementary material available at 10.1038/s41598-025-20151-1.

## Introduction

Pacific cod (*Gadus macrocephalus*), a member of the family Gadidae, is widely distributed in the northern Pacific Ocean^1^. This species is prevalent along the coastal regions of Korea and holds considerable commercial value in local fishery markets^2^. As a typical cold-water fish, Pacific cod congregates in schools in deeper waters^3,4^ and exhibits limited connectivity, with a strong signal of isolation by distance observed throughout its range in North Pacific regions^5–7^. Genetic studies in the Korean Peninsula have identified three peripheral populations of Pacific cod along the western, eastern, and southern Korean coasts^8^. Despite their seasonal spawning migration from warm- to cold-adapted populations, these populations show distinct intraspecific variations.

In demersal marine ecosystems, Pacific cod are generalist predators whose dietary habits have been extensively studied across their habitats in the North Pacific region, including Korean and Japanese waters, and the Gulf of Alaska^9^. These studies have revealed that Pacific cod typically consume teleosts^10,11^ and also ingest other crustaceans and cephalopods as primary prey items^9,12,13^. The distribution ranges of Pacific cod often overlap with those of walleye pollock (*Gadus chalcogrammus*)^14^indicating potential competition for food resources between the two species. Interspecific dietary studies on Pacific cod and walleye pollock have revealed that Pacific cod consume more benthic and larger prey items than walleye pollock^9,15^.

Several studies on the feeding patterns of Pacific cod in Korean waters have revealed distinct intraspecific variations influenced by several factors, such as habitat (location), growth (fish size), and season. As Pacific cod increases in body length, the proportion of teleosts consumed also gradually increases^12,16,17^. In addition, spatial patterns in the dietary composition of Pacific cod along the eastern, southern, and western coasts of Korea show that the southern population consumes more teleosts, whereas the eastern and western populations consume higher proportions of carid shrimps^10^. As a co-occurring species with Pacific cod in the East Sea, walleye pollock (*Gadus chalcogrammus*) also exhibited dietary patterns that varied with fish size and season; this includes ontogenetic changes from euphausiids to cephalopods and teleosts as fish size increases^18^ and an increased consumption rate of euphausiids during summer and autumn^19^. Variations in dietary patterns are prevalent among generalist predators because their prey selection is closely influenced by the locally available prey resources that are easily capturable^20^.

Studies on the dietary habitats of fish species are generally conducted using stomach content analysis, which provides high-resolution information on prey items consumed by fish species. This method can provide information on prey composition and the degree of prey selection for specific prey items because it provides taxonomic details on specific prey items^21^. Most fish dietary studies have outlined the feeding pattern characteristics of fish species via stomach content analyses^22–24^. However, the data derived from stomach content analysis reflect only the short-term dietary compositions consumed by predators a few hours prior to sampling, thereby limiting its efficacy in elucidating the long-term or continuous prey selection of fish^25^. Stable isotope analysis overcomes this limitation by offering time-integrated information on feeding habits related to prey items assimilated by consumers and providing long-term dietary selections and food pathways^26,27^. Although stable isotope analysis measures trophic niche width, identifying specific prey items contributing to that niche width remains challenging. Consequently, integrating stomach content analysis with stable isotope analysis enhances the interpretation of dietary studies on fish and complements taxonomic data with insights into trophic niche dynamics^28–30^. Several studies have also reported on the feeding ecology of cod species through the combined use of stomach content and stable isotope analyses^15,29^.

In this study, we aimed to investigate the variations in prey items and trophic niches in relation to fish size, study location, and season in the diet of Pacific cod. More specifically, the objectives of this study were to (1) demonstrate the overall prey composition of Pacific cod; (2) quantify dietary changes associated with growth in the two regions during the low- and high-temperature seasons; and (3) analyze differences in trophic level relative to body size and region. Exhibiting the dietary utilization of commercially important fish species is a valuable approach from ecological and fishery perspectives, as it provides insights into the mechanisms of ecological interactions with other fish species, including food competition and resource partitioning^26^. The results of this study can enhance our understanding of the influences of environmental changes on the diets and trophic levels of predatory fish, such as changes in water temperature and prey availability, thereby supporting sustainable fishery management and conservation.

## Materials and methods

### Study area

**Fig. 1:**
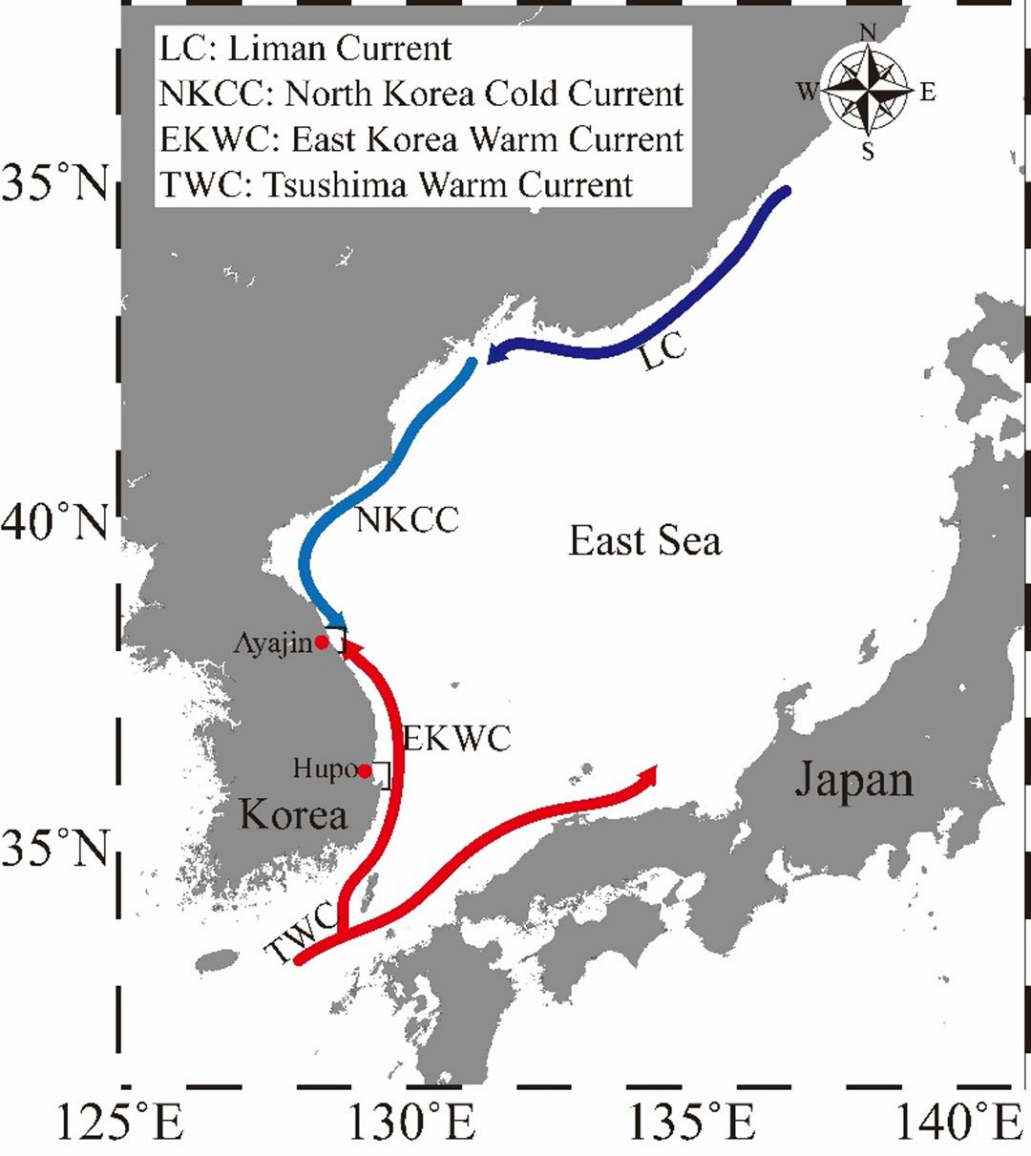
Study area located in the eastern waters off the Korean Peninsula, delineated by the enclosed boxes. The map was generated using Ocean Data View (ODV) version 5.7.0 (available at https://odv.awi.de) and edited using Adobe illustrator on desktop version 28.0.

Sampling was conducted at two latitudinal locations along the mid-eastern coast of Korea (Fig. 1), situated in the southwestern region of the East Sea (also known as the Sea of Japan). Two study sites (northern Ayajin and southern Hupo) were selected to examine latitudinal changes in the prey items of Pacific cod based on the path of the sub-currents in the East Sea. The Hupo area (36.66˚N, 129.63˚E) is located on the path of the East Korea Warm Current, whereas the Ayajin area (38.27˚N, 128.68˚E) is positioned on the boundary between the East Korea Warm Current and North Korea Cold Current.

### Sampling

Pacific cod samples were collected seasonally over five years (from 2018 to 2022) using bottom gill nets (75 m in length, 2 m in height, and 90 mm mesh) at depths ranging from 50 to 110 m at two locations (Ayajin and Hupo). In total, 261 specimens of Pacific cod were collected from Ayajin and 160 from Hupo. Because Pacific cod catches in each seasonal sampling were very small, seasonal samples were aggregated by dividing into cold (from December to March) and warm (from June to August) seasons across five years (Table S1). Conductivity, temperature, and depth (CTD) measurements were conducted simultaneously in each fish sampling occasion (except June in 2019 and 2021 at both sites; Table S1) using an onboard SBE19 plus SeaCAT profiler CTD (Sea-Bird Scientific, WA, USA) to determine the water temperature at which the Pacific cod was caught. Upon capture, samples were promptly packed on ice and transported to the laboratory for further analyses, including stomach content and stable isotope analyses. Specific measurements were taken for each individual, including the total length (TL) to the nearest millimeter and the wet weight to the nearest 0.1 g. The stomachs of all specimens were removed, and the contents were preserved in a solution containing 95% (v/v) isopropanol for at least 24 h. Dorsal muscle tissues were removed from all samples and preserved in a deep freezer until stable isotope analyses were performed. Fish samples used for dietary analyses were obtained from commercially available sources. As these samples were caught for commercial purposes, no ethical approval was required for this study.

### Stomach content analysis

The prey items of Pacific cods were analyzed by dissecting the stomachs between the esophagus and the intestine. The prey items were identified as accurately as possible to the species level and grouped into functional prey categories, generally classified at the class or order levels using a stereomicroscope (Leica EZ4E, Leica Microsystems, Wetzlar, Germany,). Dietary data were quantified through frequency of occurrence (%F = 100 × A_*i*_ × N^−1^) and weight percentage (%W = 100 × W_*i*_ × W_T_^−1^), where A_*i*_ is the number of fish consuming prey item *i*, N is the total number of fish examined (excluding those with empty stomachs), W_*i*_ is the weight of prey item *i*, and W_T_ is the total weight of all prey items within each individual stomach.

Cumulative prey curves were calculated for each species to assess whether an adequate number of stomachs were analyzed to characterize the diets^31^. Dietary data were randomized 100 times, and the cumulative number of new prey taxa was recounted for each randomization. As visual examination of prey curves for an asymptote is deemed unreliable, the slope of the linear regression (b) calculated from the last five subsamples validated the sample size, with b ≤ 0.05 indicating an acceptable leveling of the prey curves for dietary analyses^32^. The analysis was performed using R version 4.3.2 and the “vegan” package^33^.

Pacific cod diets were analyzed for the effects of fish size, study location, and season. For convenience and easy interpretation of results, size categories were classified as small (14.0–34.9 cm) and large (35.0–69.0 cm) based on the median size of the collected body size distribution and their age class of 2 year^34^. The two seasons were classified as cold (November to February) and warm (June to August). In fish dietary analysis, gravimetric or volumetric diet data most accurately reflect the relative importance of each prey taxon, especially when prey items of various sizes are consumed^35^. Additionally, ecological analyses employing a taxonomic hierarchy above the species level mitigate the influence of environmental variables on prey composition determination and enhance the visualization of prey composition outcomes^36^. Therefore, subsequent multivariate analyses of dietary patterns were performed using the weight percentage data of the prey taxa.

Dietary weight data were square-root transformed to mitigate the potential excessive dominance of main dietary components. A Bray–Curtis similarity matrix was constructed and subjected to a series of three-way permutational multivariate analyses of variance (PERMANOVA) to assess variations in dietary compositions in relations to fish size, season and study location. PERMANOVA assigns varying magnitudes of component of variation (COV) to the main factors and any three- or two-way interactions between a combination of the main factors included in the chosen comparison. The larger COV indicates the greater the influence of a particular factor or interaction term on the structure of the data^37,38^. The matrix was then visualized using a principal coordinate analysis (PCO) plot of eight groups based on fish size, study location, and season. The correlation coefficients between each factor and the PCO axis were used as evidence of prey item contributions to observed differences. All PERMANOVAs were run with 999 permutations of the residuals based on a reduced model employing a type-3 (partial) sum of squares. Statistical analyses were performed using PRIMER v7 software with the PERMANOVA add-on module^37,39^.

### Stable isotope analysis

For stable isotope analysis, 50 randomly selected individuals were analyzed in this study. Before performing stable isotope analysis, each muscle sample was subjected to oven drying at 60 °C for 48 h and then ground into a powder using a mortar and pestle. The ground samples, weighing approximately 1 mg, were placed in tin capsules. The ratios of stable carbon and nitrogen isotopes were analyzed using a continuous flow-through mass spectrometer (Isoprime CF-IRMS; Micromass, UK) connected to an elemental analyzer. The results were expressed in relation to the Pee Dee Belemnite and atmospheric N_2_ standards for δ^13^C and δ^15^N, respectively, utilizing the equation X (‰) = [(R_sample_/R_standard_) − 1] × 1000, where X represents either δ^13^C or δ^15^N, and R signifies the mass ratio of the heavy-to-light stable isotopes (^13^C/^12^C or ^15^N/^14^N) for the sample or standard, respectively. A three-way analysis of variances (ANOVAs) was performed to assess differences in the mean stable isotope values (δ^13^C and δ^15^N) among fish species. Various metrics were calculated using carbon and nitrogen stable isotope values from each sample to determine the trophic niche area of the fish species. These metrics included the total area (TA), standard ellipse area (SEA)^40^ and corrected standard ellipse area (SEAc) of the convex hull^41^. Although the TA is highly sensitive and increases exponentially with the number of samples, the SEA accurately represents the trophic niche. To account for potential underestimation due to small sample sizes, the SEAc was used to measure and compare the trophic niche widths of the fish species^41^. For this purpose, a more robust SEAc calculation method was employed, and the Stable Isotope Bayesian Ellipses in the R (SIBER) package within R software version 4.3.2 was utilized in this process.

## Results

### Vertical trends of water temperature in Northern and Southern sites

**Fig. 2:**
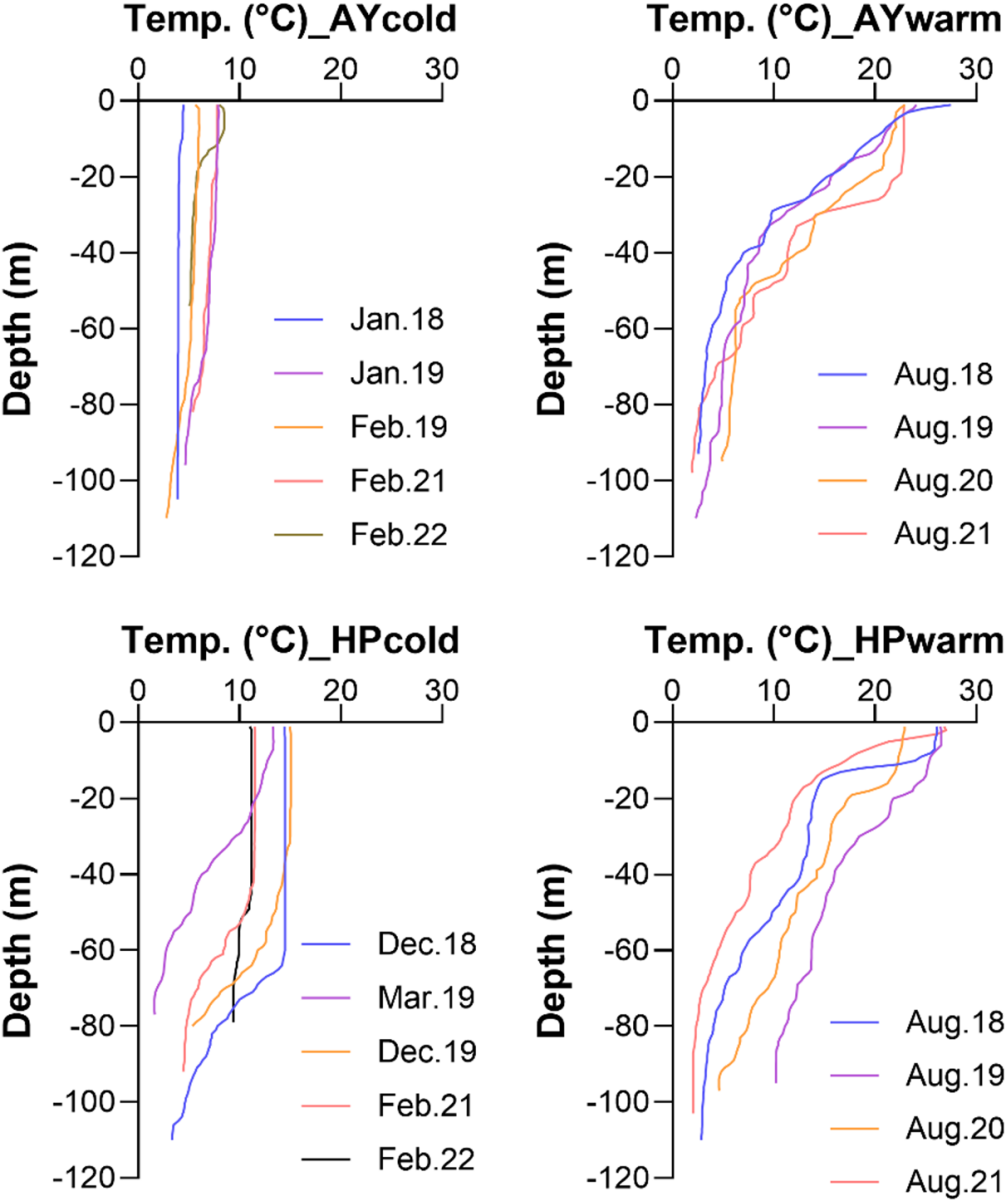
Vertical distributions of water temperature profiles during cold and warm months at Ayajin (AY) and Hupo (HP)

Water temperature exhibited distinct seasonal and depth-related patterns at both study sites (Fig. 2). Sea surface water temperatures ranged from 4.4 to 24.7 °C and 11.0 to 26.8 °C in Ayajin and Hupo, respectively. At Ayajin, temperatures in the shallow depth layer (~ 50 m) sharply declined compared to those in the surface layer during the warm season over four years, whereas those in the cold season remained consistently low across all depths (below 7.9 °C). In contrast, during the cold season at Hupo, temperatures in the shallow depth layer were higher than those at Ayajin and remained relatively constant level down to the depth of approximately up to 70 m except in March 2019. Similarly to Ayajin, vertical profiles of water temperature during warm season exhibited sharp declines from the surface to a depth of approximately 40 m, although overall temperatures varied between observation years. Consequently, water temperatures at depths from 50 to 110 m, where Pacific cod were caught, exhibited similar ranges between cold and warm seasons at each site; however, the trends were slightly higher at Hupo (1.6–14.5 °C in cold season and 2.0–14.8 °C in warm season) than those at Ayajin (2.3–7.0 °C in cold season and 2.0–8.6 °C in warm season).

### General dietary composition

In total, 421 Pacific cod samples (261 from Ayajin and 160 from Hupo) were analyzed during the study period. The body length range of the Pacific cod collected was between 14.0 and 69.0 cm TL (Fig. S1). The cumulative prey curves for the small- and large-sized classes across the Ayajin and Hupo regions demonstrated that sufficient sample sizes were obtained for the dietary analyses (Fig. S2).

Fifteen identifiable prey taxa were found in the stomachs of Pacific cod (Table 1). At Ayajin, teleosts had the highest frequency of occurrence (%F = 46.3%) and weight contribution (%W = 42.4%) of Pacific cod prey items. Amphipods and cephalopods accounted for 18.7% and 17.0% of secondary prey items by weight contribution, respectively. The main prey item of Pacific cod in the Hupo region was teleosts, which accounted for 44.5% in frequency of occurrence and 40.4% in weight contribution, followed by carid shrimps and cephalopods, with frequencies of occurrence of 30.0% and 26.4% and weight contributions of 24.8% and 20.7%, respectively.

**Table 1:** Frequency of occurrence (%F) and weight contribution (%W) of prey items in the diets of Pacific cod (*Gadus macrocephalus*) at Ayajin and hupo in the eastern coast of Korea.

		Ayajin		Hupo	
Taxa	Prey items	%F	%W	%F	%W
**Nematoda**	**Unidentified**	**5.1**	**< 0.1**	**1.8**	**0.7**
**Crinoidea**	**Unidentified**	**0.6**	**0.2**	**-**	**-**
**Polychaeta**	**Total**	**1.1**	**1.1**	**2.7**	**2.1**
	*Cirratulida* sp.	0.6	< 0.1	-	-
	Flabelligeridae sp.	0.6	0.5	-	-
	Unidentified	0.6	0.6	2.7	2.1
**Gastropoda**	**Total**	**1.1**	**0.6**	-	-
	*Calliostoma unicum*	0.6	< 0.1	-	-
	Unidentified	0.6	0.6	-	-
**Bivalvia**	**Total**	**1.1**	**0.4**	**1.8**	**0.1**
	Tellinidae sp.	0.6	< 0.1	1.8	0.1
	Unidentified	0.6	0.4	-	-
**Cephalopoda**	**Total**	**22.0**	**17.0**	**26.4**	**20.7**
	*Berryteuthis magister*	1.7	1.7	-	-
	Octopus sp.	-	-	1.8	1.8
	Sepiolidae spp.	0.6	0.1	1.8	1.7
	*Todarodes pacificus*	0.6	0.6	-	-
	*Watasenia scintillans*	4.0	3.5	2.7	2.7
	Unidentified	15.3	11.3	20.0	14.4
**Amphipoda**	**Total**	**27.1**	**18.7**	**3.6**	**1.1**
	*Anonyx* sp.	4.0	3.1	-	-
	Caprellidae spp.	0.6	0.4		-
	Gammaridea spp.	1.7	1.1	0.9	< 0.1
	Hyperiidae spp.	16.9	11.8	2.7	1.1
	Lysianassidae sp.	0.6	0.5	-	-
	Oedicerotidae sp.	2.3	1.7	-	-
	*Paraphronima gracilis*	1.1	< 0.1	**-**	**-**
	*Peramphithoe* sp.	0.6	< 0.1	-	-
**Euphausiacea**	**Unidentified**	**14.1**	**10.1**	**0.9**	**0.9**
**Mysidacea**	**Unidentified**	**3.4**	**1.3**	**11.8**	**7.5**
**Caridea**	**Total**	**7.3**	**4.9**	**30.0**	**24.8**
	*Crangon hakodatei*	-	-	0.9	0.9
	Crangonidae spp.	1.1	0.6	5.5	4.8
	*Eualus middendorffi*	1.1	1.0	0.9	0.9
	*Lebbeus polaris*	0.6	0.5	**-**	**-**
	*Neograngon communis*	4.0	2.2	13.6	10.4
	*Pandalus borealis*	0.6	0.6	**-**	**-**
	*Paracrangon echinatus*	-	-	0.9	0.3
	*Plesionika ortmanni*	-	-	0.9	0.4
	*Spirontocaris spinus*	**-**	**-**	0.9	0.5
	Unidentified	**-**	**-**	7.3	6.5
**Paguroidea**	**Total**	**0.6**	**0.5**	**0.9**	**0.7**
	*Pagurus trigonocheirus*	0.6	0.5	-	-
	*Dardanus arrosor*	**-**	**-**	0.9	0.7
**Brachyura**	**Total**	**1.1**	**0.2**	**1.8**	**0.9**
	*Chionoecetes opilio*	-	-	0.9	0.8
	Unidentified	1.1	0.2	0.9	0.2
**Echinoidea**	**Unidentified**	-	-	**0.9**	**0.2**
**Ophiuroidea**	**Total**	**1.1**	**< 0.1**	-	-
	*Ophiarachnella gorgonia*	1.1	< 0.1	-	-
**Teleostei**	**Total**	**46.3**	**42.4**	**44.5**	**40.4**
	Agonidae sp.	1.7	1.6	-	-
	*Arctoscopus japonicus*	8.5	8.3	-	-
	*Clupea pallasii*	0.6	0.6	3.6	3.6
	*Engraulis japonica*	0.6	0.1	1.8	1.8
	Gadidae spp.	1.7	1.5	-	-
	*Glyptocephalus stelleri*	0.6	< 0.1	-	-
	*Hypoptychus dybowskii*	1.1	1.1	0.9	0.9
	Pleuronectidae sp.	0.6	0.1	-	-
	Unidentified	32.2	29.1	38.2	34.0
**Other materials**	**Unidentified**	**5.6**	**2.4**	**0.0**	**0.0**

### Spatial, temporal, and size-dependent changes in dietary composition

**Fig. 3:**
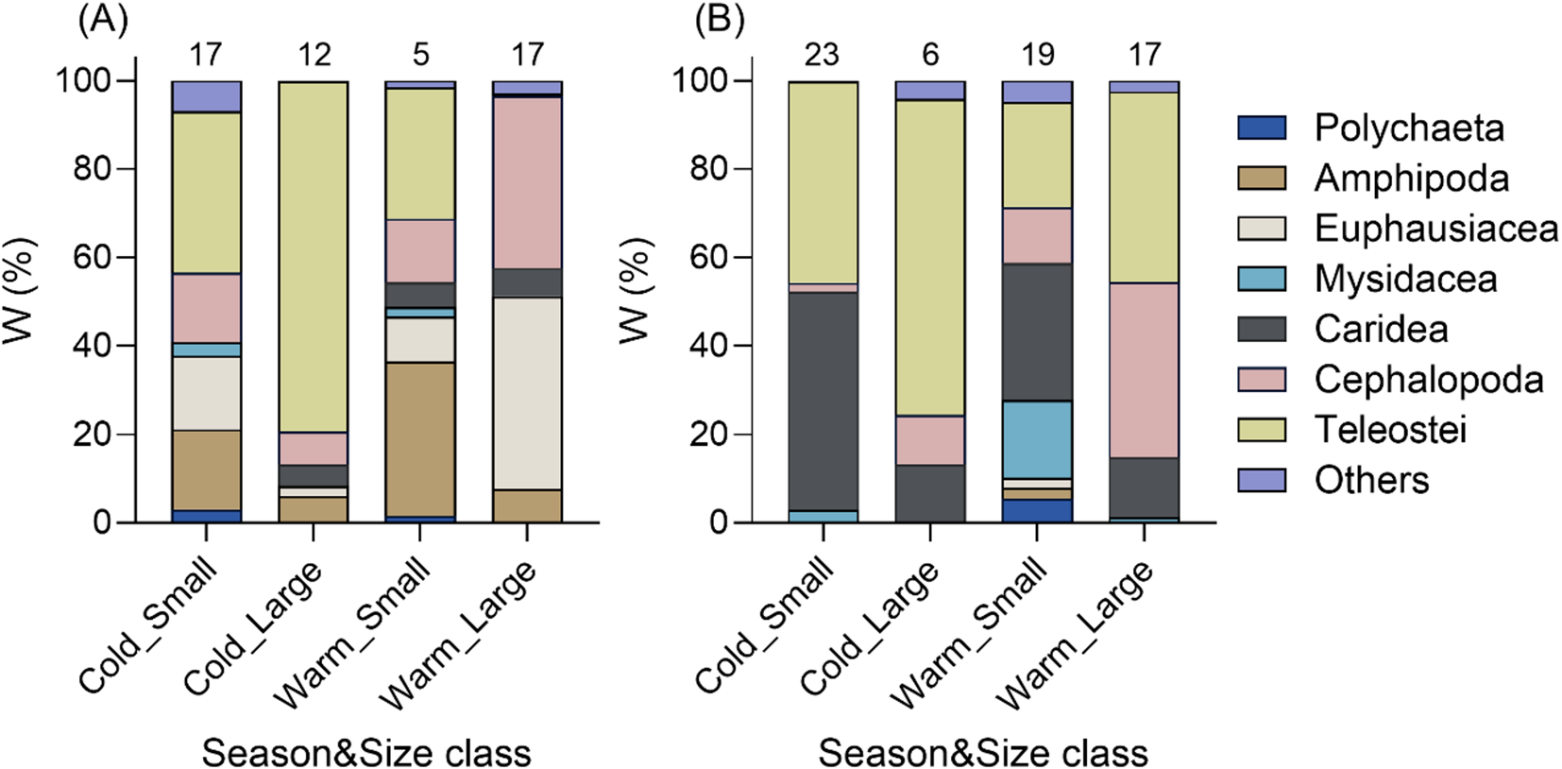
Dietary contributions of prey taxa categorized by size class over seasons at Ayajin (**A**) and Hupo (**B**) in the diets of Pacific cod (*Gadus macrocephalus*). The other categories include bivalves, brittle stars, crabs, crinoids, debris, echinoids, gastropods, hermit crabs, and nematodes

Analysis of the gravimetric dietary data revealed distinct spatiotemporal and size-related patterns in the dietary composition of Pacific cod (Fig. 3). At Ayajin, teleosts displayed the highest weight contributions to the diets of both small- and large-sized classes of Pacific cod during the cold season (79.3% and 36.5%, respectively). Amphipods (18.1%), cephalopods (15.8%), and euphausiids (16.6%) collectively constituted the next most abundant dietary components of the small-sized class of Pacific cod. During the warm season, the proportions of euphausiids (43.5%) and amphipods (35.0%) were the highest in the diets of the large- and small-sized classes of Pacific cod, respectively. Teleosts constituted the main dietary component of Pacific cod caught at Hupo for large-sized Pacific cod, regardless of season. Card shrimp were important prey items for small-sized Pacific cod during both cold and warm seasons. In addition to teleosts, cephalopods were the next most important prey item for the large-sized Pacific cod during the warm season.

### Dietary pattern data analysis

**Fig. 4:**
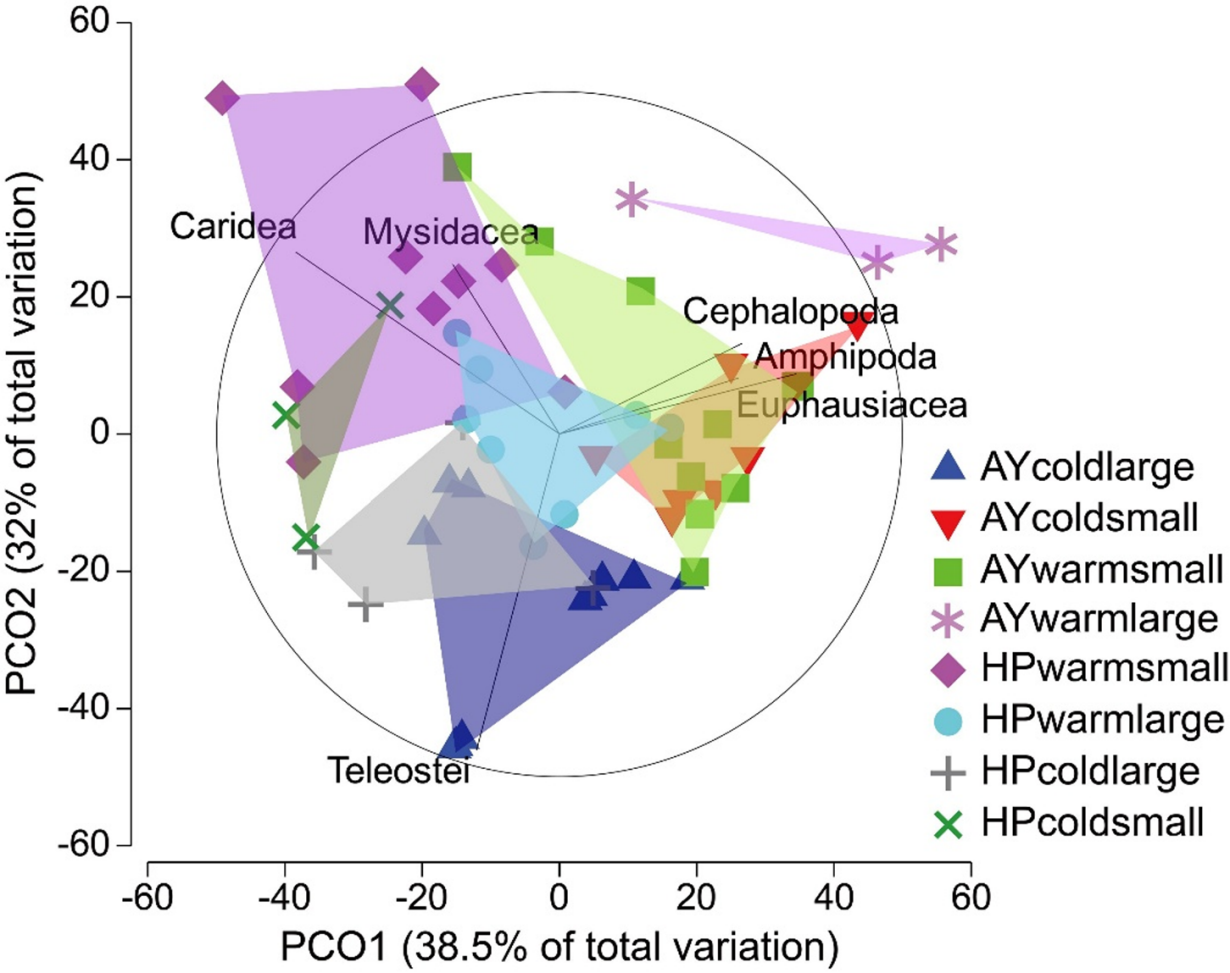
Principal coordinates analysis (PCO) plot derived from the dietary composition of Pacific cod (*Gadus macrocephalus*). The vectors are plotted to illustrate the strength and direction of the correlations of prey taxa among site, season, and size relative to the PCO axes, with the cycle indicating a correlation of 1. Shaded polygon areas indicate the convex hulls of total area for the eight feeding groups.

The three-way PERMANOVA results indicated significant two-way interactions between site and size, season and size and a three-way interaction among all three factors; however, the two-way interaction between site and season was not significant (Table 2). Principal coordinate analysis revealed distinct separations in the samples, consistent with the three-way PERMANOVA results (Fig. 4). Samples from Ayajin were located on the lower and right side of the PCO plot, whereas those from Hupo were concentrated in the upper and left side of the plot. The two size classes and two seasons distinguished themselves within both Ayajin and Hupo. Samples from the cold season were distributed on the lower side of the plot, whereas those from the warm season were located in the middle or upper area. Teleosts were key in distinguishing large-sized Pacific cod in Ayajin during the cold season, whereas carid shrimps and mysids were characteristic of the diets of small-sized Pacific cod in Hupo throughout all seasons (Fig. 4). Additionally, cephalopods, amphipods, and euphausiids collectively made up the diets of the small-sized class of Pacific cod in Ayajin (Fig. 3).

**Table 2:** Mean of squares, pseudo-F ratios (F), significance levels (P-Values), and components of variation (COV) for a series of PERMANOVA tests, employing the Bray–Curtis similarity matrix derived from the mean percentage weight contributions of the various prey taxa to the stomach contents of Pacific Cod (*Gadus macrocephalus*). Bold letters indicate statistical significance at *P* = 0.05.

Source	df	MS	Pseudo-F	*P*(perm)	COV
Site	1	33,123	10.962	**0.001**	17.883
Season	1	37,089	12.274	**0.001**	19.024
Size	1	17,518	5.7976	**0.001**	12.410
Site×Season	1	5096.8	1.6868	0.145	6.640
Site×Size	1	10,405	3.4434	**0.008**	12.525
Season×Size	1	20,225	6.6933	**0.001**	19.119
Site×Season×Size	1	11,972	3.9619	**0.006**	19.502
Residual	257	3021.6			54.970

### Stable isotope signatures

The δ^13^C range was − 20.0‰ to −16.8‰, while the δ^15^N range was 10.7‰ to 15.9‰ (Table S2). The δ^13^C values ranged from − 20.0 to −17.9‰ at Ayajin and − 18.9 to −16.8‰ at Hupo. The δ^15^N values ranged from 10.7 to 13.6‰ at Ayajin and 11.6 to 15.9‰ at Hupo, indicating slightly enriched isotope values in the Hupo samples (Table S1). The three-way ANOVA results showed significant differences in both δ^13^C and δ^15^N values between the two sites and two size classes; however, no significant seasonal differences were observed (Table 3).

**Table 3:** Results of a three-way ANOVA examining the effects of site, season, and size class on carbon (δ^13^C) and nitrogen (δ^15^N) stable isotopes of Pacific cod (*Gadus macrocehpalus*). Bold letters indicate statistical significance at *P* = 0.05.

Source	Df	MS	F	*P*
**δ** ^**13**^ **C**				
Site	1	30.201	120.929	**< 0.001**
Season	1	0.192	0.767	0.386
Size	1	1.793	7.180	**0.010**
Site×Season	1	2.381	9.535	**< 0.001**
Site×Size	1	0.131	0.524	0.473
Season×Size	1	0.120	0.479	0.492
Site×Season×size	1	0.000	0.002	0.965
Residuals	43	0.250		
**δ** ^**15**^ **N**				
Site	1	43.698	51.800	**< 0.001**
Season	1	0.395	0.469	0.497
Size	1	11.356	13.462	**< 0.001**
Site×Season	1	8.079	9.577	**0.003**
Site×Size	1	0.380	0.450	0.506
Season×Size	1	0.022	0.026	0.873
Site×Season×size	1	< 0.001	< 0.001	0.998
Residuals	43	0.844		

**Fig. 5:**
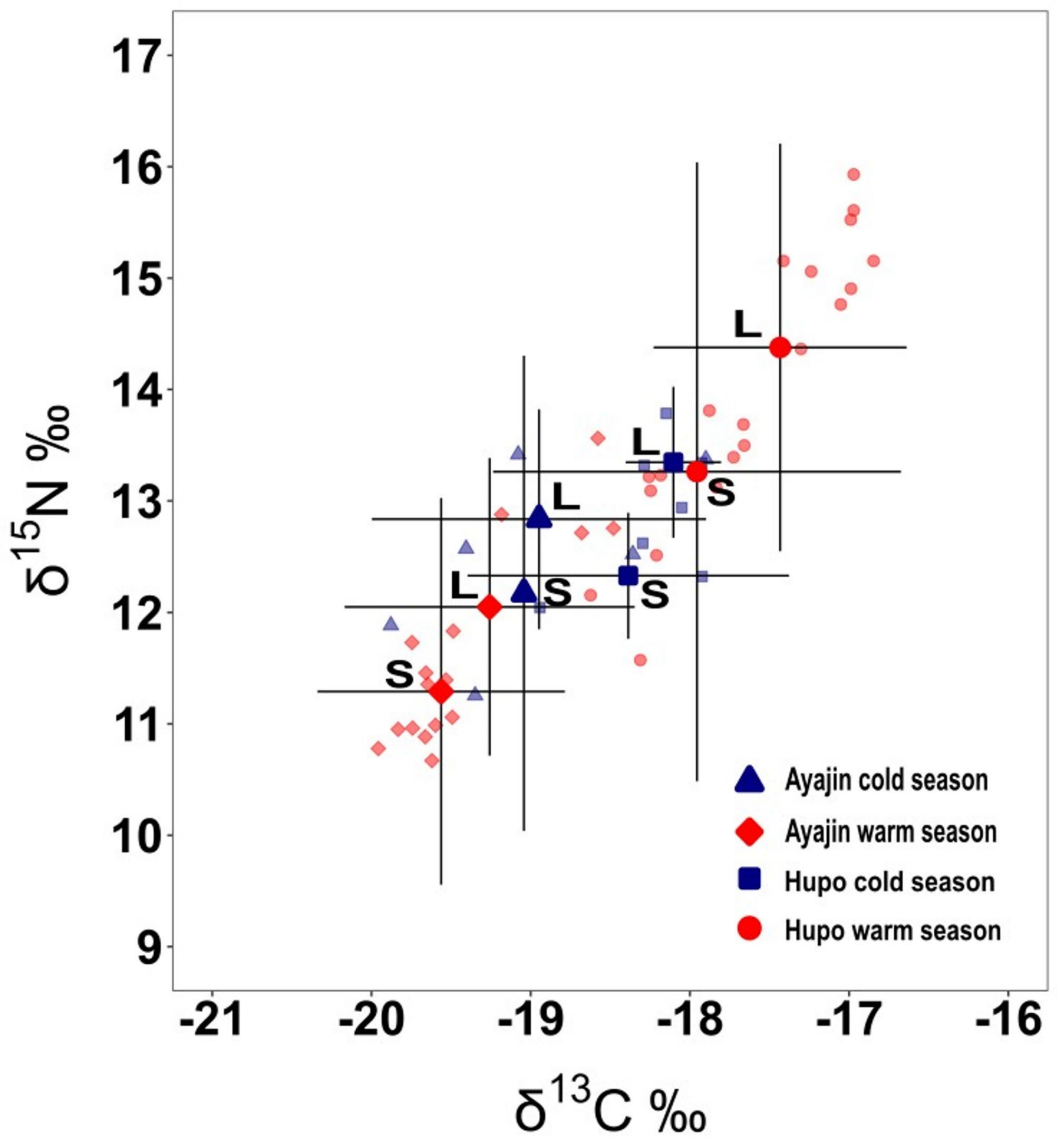
Biplot of carbon (δ^13^C) and nitrogen (δ^15^N) stable isotope values for Pacific cod (*Gadus macrocephalus*) categorized by site, season, and size class. Colored symbols represent means (± SE) for each sample, while transparent symbols denote individual values (L = large-sized class, S = small-sized class)

**Fig. 6:**
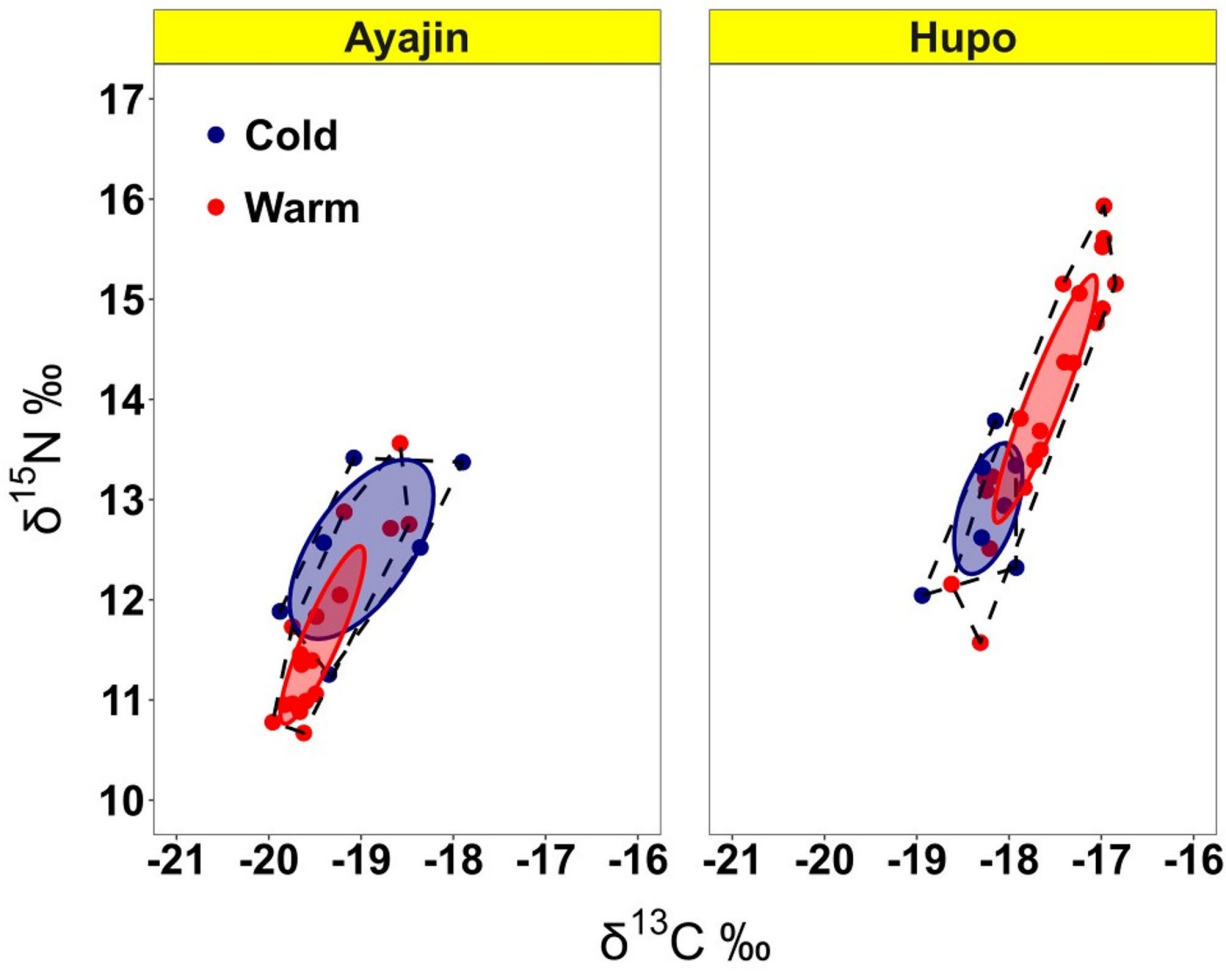
Characteristics of the trophic niche space of Pacific cod at Ayajin and Hupo during the two seasons. The solid lines depict the corrected standard ellipse areas (SEAc), while the dotted lines indicate the convex hull total areas (TA), both derived from their stable isotope values

Large-sized Pacific cod were significantly enriched in mean δ^13^C and δ^15^N values relative to small-sized individuals (three-way ANOVA, *P* < 0.05; Fig. 5). In addition, the Pacific cod samples collected from Hupo consistently exhibited higher δ^13^C and δ^15^N values than Ayjin samples regardless of season and size class. Although no significant seasonal differences were observed in both δ^13^C and δ^15^N values within each study site, warm season samples had higher δ^13^C and δ^15^N values in Hupo, whereas a contrasting trend was evident in Ayajin (Fig. 5). The TAs of isotopic values were the highest in the warm season samples at Hupo (2.148) and lowest in the cold season samples at Hupo (0.889). The SEAc values were the highest during the cold season at Ayajin (1.935) and lowest during the warm season at Ayajin (0.605) (Fig. 6).

## Discussion

Stomach content analysis typically provides direct insights into the feeding behavior and prey selection of aquatic animals, including marine fish^23^. In contrast, stable isotope signatures reveal the trophic levels of marine fish, reflecting their long-term prey choices^42^. The feeding behavior of marine fish often varies based on body size and environmental conditions, reflecting their capacity to capture prey items and the availability of food resources within their habitats^43^. Therefore, a comprehensive understanding of how environmental variables influence the dietary habits and feeding behaviors of fish species necessitates both direct observations of prey items via stomach content and trophic level analyses using stable isotope ratios to determine the functional roles of fish within an ecosystem^18,44^. Consequently, this study revealed distinct spatiotemporal trends in feeding patterns based on fish size, site, and season, as evidenced by the results of dietary compositions and stable isotope ratios.

In the Pacific cod diets, teleosts exhibited the highest frequency of occurrence and weight proportion at both sites, particularly during the cold season and within the diets of the large-sized class. Generally, Pacific cod possess relatively large mouth gaps and robust bodies, facilitating the capture of larger and high-energy prey items, such as fish and cephalopods^45^. Numerous studies have reported that teleosts constitute the primary prey items for Pacific cod^11,16,46^whereas some studies have also documented crustaceans as the preferred food items for Pacific cod inhabiting the southern coast of Korea^10,12^ and the northern Gulf of Alaska^9^. In addition, Pacific cod exhibited latitudinal differences in the specific fish prey species within their diets. Among the identifiable fish prey items, the consumption of Pacific herring (*Clupea pallasii*) was higher at Hupo than Ayajin, whereas that of Japanese sandfish (*Arctoscopus japonicus*) was found only in the diets of Pacific cod from Ayajin. For example, on the eastern coast of Korea, Japanese sandfish is the dominant demersal fish species in the mid-northern coastal region of the East Sea at a higher than latitude of 37°N, whereas Pacific herring is a common fish species in the southern area^47^. According to niche theory and optimal foraging theory, larger individuals of fish select different prey across regions because their body size influences shifts in fundamental and realized niches, while regional prey diversity and availability determine the maximization of net energy gain, thereby shaping diets through niche shifts and optimal foraging trade-offs^48–50^. Therefore, the diets of Pacific cod are indicative of locally available food resources, as they are typical opportunistic predators that adjust their feeding strategy based on the availability and encounters of prey items^11^.

In contrast, smaller specimens of Pacific cod consumed more euphausiids and amphipods at Ayajin and carid shrimps at Hupo. Euphausiids are typical cold-water marine organisms mainly inhabiting water temperatures under 10°C^51,52^, and they often serve as a food source for demersal fish species, such as Gadidae fish, in colder water conditions in the Northwest Pacific^18,53^. In addition, carid shrimps constitute the dominant epibenthic invertebrate group on the southeastern coast of Korea^54^ and contribute substantial food resources for demersal fish assemblages in this region^22^. Based on the vertical water temperature profile, water temperatures below a depth of 50 m at Ayajin, where Pacific cod was collected (Fig. 2), constantly remained below 10 °C, indicating the influence of North Korea Cold Current at the meddle-depth layer^55,56^. In contract, water temperature at Hupo often exceeded 10 °C, suggesting that the thermal conditions may be unsuitable for occurring euphausiids. These regional differences in available food resources can be attributed to regional and seasonal differences in available prey items in relation to oceanographical conditions, which may influence prey choice in the generalist diets of Pacific cod^9^.

The diets of large-sized Pacific cod show an increased proportion of cephalopods during the warm season at both sites. Among cephalopod prey species, *Watasenia scintillans* was the most abundant cephalopod prey. They are a relatively small sized and cold water squid species primarily distributed in the Northwest Pacific including the eastern coast of Korea^57^. *Watasenia scintillans* individuals also exhibit diel vertical migration, residing at depths between 300 and 400 m during the day and 20 and 60 m during the night^58^; therefore, their nighttime distributional depths highly overlap with the main habitat of the Pacific cod^3^. In addition, adult *W. scintillans* inhabit deep waters (200–600 m) for most of the year, but undertake a seasonal migration to coastal area of Northwest Pacific region (e.g., Toyama Bay) during spring and summer for spawning^59^thereby enhancing feeding opportunities for Pacific cod on *W. scintillans* in shallow nearshore region. Similarly, several demersal fish species inhabiting the eastern coastal regions of Korea exhibit increased consumption of mesopelagic squids during the warm season^60,61^.

In this study, Pacific cod individuals showed distinct differences in carbon and nitrogen stable isotope values between Ayajin and Hupo, and between small- and large-sized classes. Variations in stable isotope values reflect changes in predator diets over time and ontogeny, suggesting shifts in the dietary patterns of marine predators^62–64^. Generally, consumer bodies have different carbon and nitrogen stable isotope values depending on the type of prey items consumed^18,65^. Notably, carid shrimps are more benthic and positioned at a higher trophic level than amphipods or euphausiids, reflecting relatively higher carbon and nitrogen stable isotope values^18,62,66^. Higher carbon and nitrogen stable isotope values in Pacific cod from Hupo than in those from Ayajin may be attributed to the increased consumption of carid shrimps rather than that of amphipods or euphausiids. In addition, the higher consumption of teleosts and cephalopods in the diets of large-sized Pacific cod indicated higher trophic levels relative to smaller conspecifics at each site. As indicated earlier, stomach content and stable isotope analyses often produce differing results owing to differences in temporal resolution between the two methodologies^26^; nonetheless, our findings revealed consistent patterns in resource use differences between the two regions across both analyses. However, some seasonal deviation between isotope values and actual food intake was also observed in the cold season diets of Pacific cod at Hupo. This discrepancy may be explained by the possibility that the fish consumed mainly teleosts but did not fully assimilate these high-nutrition prey because of lowered metabolic rates during the cold season^67^. In addition, the analysis of the stable isotope ellipse area showed large standard deviations and relatively wide SEAc ranges across seasons in each region. An increased isotopic niche width during cold season in Ayajin, as reflected by larger SEA, suggests a broader dietary spectrum and greater trophic diversity. Consequently, the results suggest that the Pacific cod occupies a relatively broad trophic niche, covering more than one trophic level^41^.

In conclusion, this study examined the dietary habitats of Pacific cod commonly caught on the eastern coast of Korea using both stomach content and stable isotope analyses. Both methodologies indicated the degree of resource utilization for different prey items with respect to fish size, study location, and season. Notably, larger individuals of Pacific cod consumed more teleosts and cephalopods—especially during the cold and warm seasons, respectively—while smaller individuals relied more on carid shrimps, amphipods, and euphausiids. Corresponding differences in stable isotope values (δ¹³C and δ¹⁵N) revealed higher trophic positions in larger individuals and at Hupo, primarily due to increased consumption of higher-trophic-level prey such as teleosts or carid shrimps. Overall, the results of this study contribute to the understanding of differences in trophic relationships between predators and their prey items based on the environmental conditions of the benthic ecosystems of the East Sea of Korea. Because cold water prey items such as euphausiids are highly vulnerable to rising water temperature in temperature oceans^18^, future and continuous research will be necessary to predict the potential northward shift in the distribution of specific prey species, and to assess how such changes, in conjunction with temperature rise models, may influence the diet and habitat selection of Pacific cod. As Pacific cod is common and commercial species in the East Sea off Korea, examining their dietary habits and trophic ecology is particularly essential for understanding the biological interactions within benthic ecosystems, and finally can be transformed into suggestions for resource conservation or fishing management.

## Supplementary Information

Below is the link to the electronic supplementary material.

Supplementary Material 1

## Data Availability

The datasets used and/or analyzed during the current study are available from the corresponding author on reasonable request.
