# Supplementary material for: Combined stomach content and stable isotope analyses revealed variations in trophic ecology of pacific cod in the east sea
Source: Sci Rep. 2025 Oct 17;15:36339. doi: 10.1038/s41598-025-20151-1 (PMC12534371; doi:10.1038/s41598-025-20151-1)
Supplement: Supplementary file 1 — Supplementary Material 1 [file 41598_2025_20151_MOESM1_ESM.doc]

**Supplementary materials**


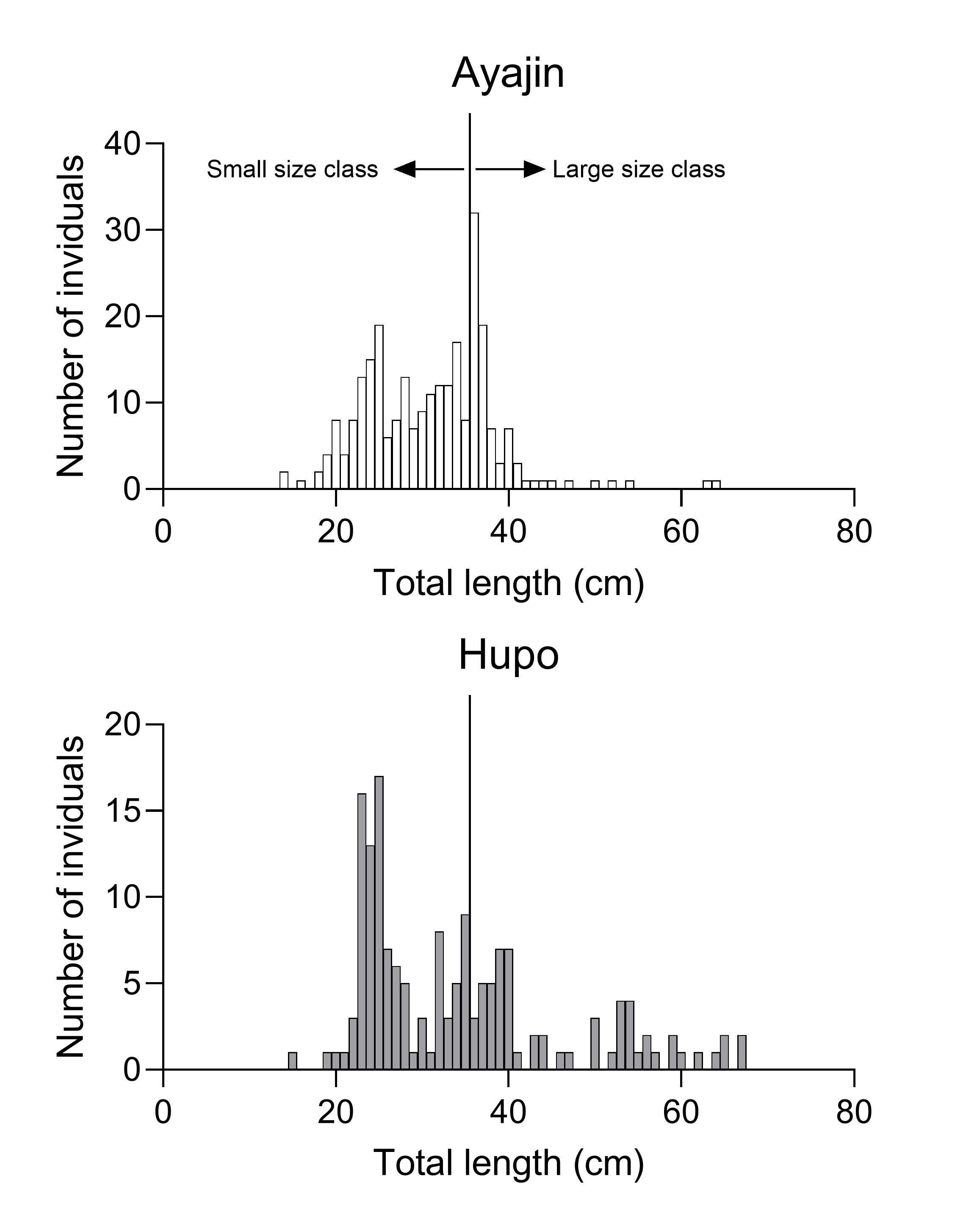


**Figure S1**. Cumulative prey curves and confidence interval of 95% upper and lower with b values through the last five subsamples for Pacific cod with respect to site and size.


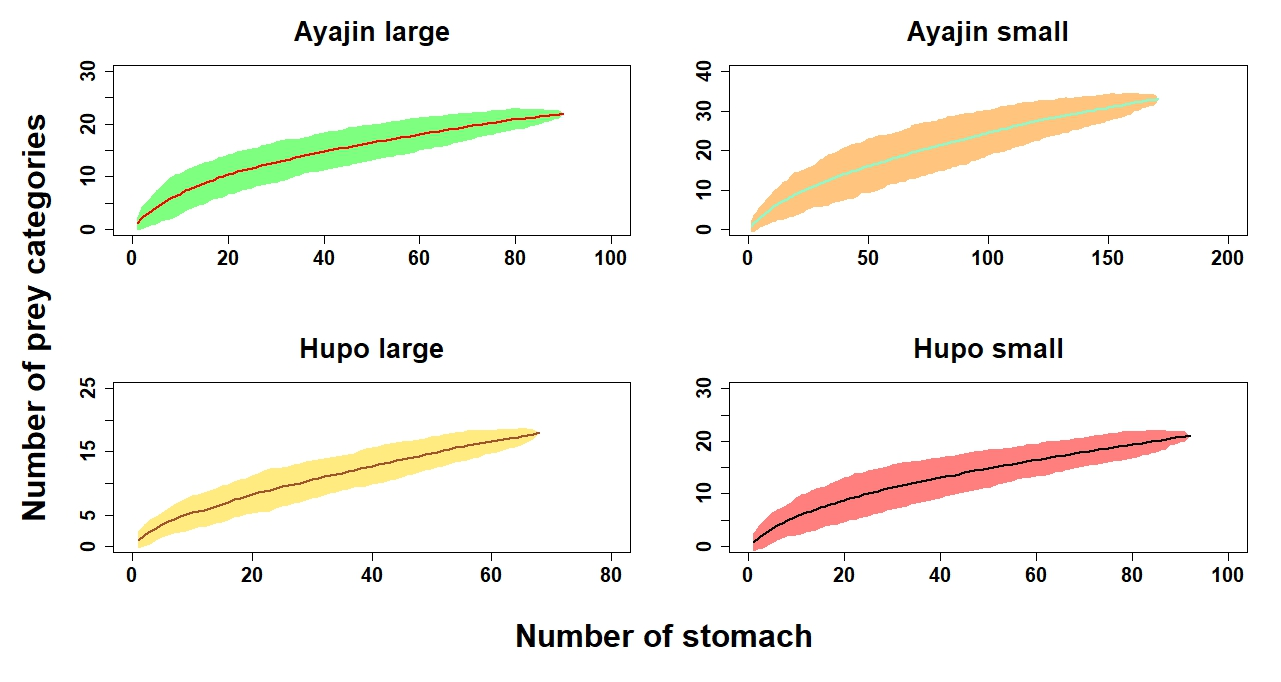


**Figure S2**. Cumulative prey curves and confidence interval of 95% upper and lower with b values through the last five subsamples for Pacific cod with respect to site and size.

**Table S1.** Specific information of sampling year, month, number of Pacific cod catch (N) and CTD observation in each study site

| Study site | Year | Cold season | | | Warm season | | |
| --- | --- | --- | --- | --- | --- | --- | --- |
|  |  | Month | N | CTD | Month | N | CTD |
| Ayajin | 2018 | January | 57 | ○ | August | 57 | ○ |
|  | 2019 | January | 8 | ○ | August | 40 | ○ |
|  |  | February | 30 | ○ |  |  |  |
|  | 2020 | - | - | - | August | 13 | ○ |
|  | 2021 | February | 5 | ○ | June | 11 | × |
|  |  |  |  |  | August | 30 | ○ |
|  | 2022 | February | 10 | ○ | - | - | - |
| Hupo | 2018 | December | 15 | ○ | August | 82 | ○ |
|  | 2019 | March | 7 | ○ | June | 10 | × |
|  |  | December | 3 | ○ | August | 12 | ○ |
|  | 2020 | - | - | - | August | 16 | ○ |
|  | 2021 | February | 4 | ○ | June | 2 | × |
|  |  |  |  |  | August | 6 | ○ |
|  | 2022 | February | 3 | ○ | - | - | - |

**Table S2.** The range and mean ± SD of stable carbon (δ^13^C) and nitrogen (δ^15^N) isotope values of Pacific cods (*Gadus macrocephalus*) caught in the two sites (Ayajin and Hupo) in each season and size class

| Season | Size class | Ayajin | | | | | | Hupo | | | | | |
| --- | --- | --- | --- | --- | --- | --- | --- | --- | --- | --- | --- | --- | --- |
|  |  | δ^13^C | | | δ^15^N | | | δ^13^C | | | δ^15^N | | |
|  |  | Min | Max | Mean±SD | Min | Max | Mean±SD | Min | Max | Mean±SD | Min | Max | Mean±SD |
| Cold | Small | **19.9** | **17.9** | **-19.0±1.0** | **11.3** | **13.4** | **12.2±1.1** | **18.9** | **17.9** | **-18.4±0.5** | **12.0** | **12.6** | **12.3±0.3** |
|  | Large | **19.4** | **18.4** | **-19.0±0.5** | **12.5** | **13.4** | **12.9±0.5** | **18.3** | **17.9** | **-18.1±0.2** | **12.9** | **13.8** | **13.3±0.3** |
| Warm | Small | **20.0** | **18.6** | **-19.6±0.4** | **10.7** | **13.6** | **11.3±0.9** | **18.6** | **17.1** | **-18.0±0.7** | **11.6** | **15.5** | **13.3±1.4** |
|  | Large | **19.7** | **18.5** | **-19.3±0.5** | **11.0** | **12.9** | **12.1±0.7** | **18.2** | **16.8** | **-17.4±0.4** | **13.1** | **15.9** | **14.4±0.9** |
